# GALNT6 promotes breast cancer metastasis by increasing mucin-type O-glycosylation of α2M

**DOI:** 10.18632/aging.103349

**Published:** 2020-06-18

**Authors:** Chang Liu, Zhi Li, Lu Xu, Yu Shi, Xiaojie Zhang, Sha Shi, Kezuo Hou, Yibo Fan, Ce Li, Xiaoxun Wang, Lu Zhou, Yunpeng Liu, Xiujuan Qu, Xiaofang Che

**Affiliations:** 1 Department of Medical Oncology, The First Hospital of China Medical University, Shenyang 110001, China; 2 Key Laboratory of Anticancer Drugs and Biotherapy of Liaoning Province, The First Hospital of China Medical University, Shenyang 110001, China; 3 Department of Internal Medicine, Cancer Hospital of China Medical University, Liaoning Cancer Hospital and Institute, Shenyang 110042, China

**Keywords:** GALNT6, α2M, breast cancer, O-glycosylation, metastasis

## Abstract

Breast cancer is the most lethal malignancy in women. N-acetylgalactosaminyltransferase 6 (GALNT6) is an enzyme which mediates the initial step of mucin-type O-glycosylation, and has been reported to be involved in mammary carcinogenesis. However, the molecular mechanism of GALNT6 in breast cancer metastasis has not been fully explored. In this study, based on online database analyses and tissue microarrays, the overall survival (OS) of breast cancer patients with high expression of GALNT6 was found to be shorter than those with low expression of GALNT6. Also, high GALNT6 expression was positively correlated with advanced pN stage and pTNM stage. GALNT6 was shown to be able to promote the migration and invasion of breast cancer cells, and enhance the level of mucin-type O-glycosylation of substrates in the supernatants of breast cancer cells. Qualitative mucin-type glycosylomics analysis identified α2M as a novel substrate of GALNT6. Further investigation showed that GALNT6 increased O-glycosylation of α2M, and the following activation of the downstream PI3K/Akt signaling pathway was involved in the promotion of migration and invasion of breast cancer cells. This study identified a new substrate of GALNT6 and provides novel understanding of the role of GALNT6 in promoting metastasis and poor prognosis in breast cancer.

## INTRODUCTION

Breast cancer is the most common malignancy and the leading cause of cancer-related death in women, with an increasing incidence rate of 3.1% per year [1]. Although post-operative comprehensive treatment effectively prolongs the survival rate of the patients, 10-15% of breast cancer patients have progressive disease and distant metastasis within three years after initial diagnosis [2, 3]. To further improve disease outcomes, there is an urgent need to explore the molecular mechanisms of proliferation and metastasis in breast cancer.

Glycosylation is a common post-translational modification which is involved in a variety of biological process such as intercellular communication [4], signal transduction [5] and maintenance of protein stability [6]. It is also known that glycosylation is related to malignant phenotypes, and that some glycoproteins such as CA125 and CA19-9 have utility as tumor biomarkers [7–12]. Cancer associated-glycoproteins are predominantly ascribed to transcriptional dysregulation of glycosyltransferases [13]. Mucin-type O-glycosylation is a diverse form of glycosylation that is initiated by a family of 20 polypeptides named N-acetylgalactosaminyltransferases (GALNTs). GALNTs catalyze the transfer of GalNAc to serine/threonine residues of the substrate protein [14]. GALNTs are differentially expressed in malignant tumors and influence multiple key processes in tumorigenesis and progression, including cell proliferation [15], immune evasion [16], and metastasis [17]. GALNTs have differential but partly overlapping substrate specificities, which make the relationship between mucin-type O-glycosylation catalyzed by distinct GALNTs and tumor progression unclear [18].

Growing studies have reported that abnormal mucin-type O-glycosylation mediated by GALNTs can promote proliferation, survival and metastasis in breast cancer cells. GALNT6, as one of key enzymes catalyzing the mucin-type O-glycosylation, is regarded as the most prominent breast cancer-associated GALNT. It has been reported that the mRNA level of GALNT6 is positively correlated with bone marrow infiltration in patients with breast cancer [19]. GALNT6 has also been shown to promote tumorigenesis and metastasis by catalyzing mucin-type O-glycosylation-mediated stabilization of MUCl and fibronectin (FN) in breast cancer cells [20, 21]. In addition, GALNT6-mediated mucin-type O-glycosylation can increase nuclear translocation of estrogen receptor alpha (ERα) in breast cancer [22]. These findings suggest that GALNT6 might play an important role in the prognosis of breast cancer. However, only limited substrates of GALNT6 and their mechanisms in the development of breast cancer have been reported. It is necessary to identify additional GALNT6 substrates, and to clarify the specific molecular mechanisms of GALNT6 in breast cancer metastasis.

In this study, we analyzed the effect of GALNT6 on the survival of breast cancer patients using online databases and breast cancer clinical samples, and investigated the role of GALNT6 in the migration and invasion of breast cancer cells. Furthermore, we identified α2-macroglobulin (α2M) as a GALNT6 substrate and showed that GALNT6-mediated α2M glycosylation could promote metastasis via the AKT signaling pathway in breast cancer cells. This study provides novel understanding of GALNT6 in promoting metastasis and poor prognosis of breast cancer.

## RESULTS

### GALNT6 was overexpression in breast cancer

**Figure 1:**
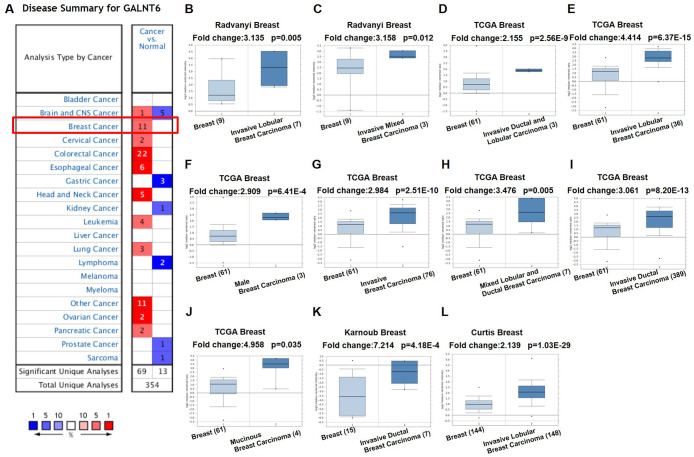
**mRNA level of GALNT6 in different tumor types in the Oncomine database.** (**A**) This graphic presentation showed the number of datasets with statistically significant mRNA high expression (red) or low expression (blue) of GALNT6 (cancer *vs.* normal tissue). The fold change was defined as 2 and *p*-value threshold was set up at 0.05. The number in each cell represents the number of analyses that met the threshold within the analysis and cancer types. The gene rank was analyzed by percentile of target gene in the top of all genes as measured in each research. Cell color is determined by the best gene rank percentile for analyses within the cell. Gene rank: Genes are ranked by their p-value for every analysis. (**B**–**L**) Comparison of GALNT6 expression in normal and breast cancer tissues.

The expression of GALNT6 in 20 types of cancers was evaluated using the Oncomine database. Search results showed that the expression of GALNT6 in breast cancer tissues was 2.139 - 7.214 fold higher than that in normal tissues in 11 out of 45 analyses (analyses refer to different analyses with multiple samples). Among the 11 analyses, 9 analyses contained patients with invasive ductal and/or lobular breast carcinoma, one analysis contained patients with mucinous breast carcinoma and one analysis contained patients with male breast carcinoma (Figure 1, Table 1, and Supplementary Table 1). These results indicated that GALNT6 might play an essential role in breast cancer progression.

**Table 1:** GALNT6 differential transcript expression in human breast cancer from multiple studies in the Oncomine database.

**Study**	**Comparison (specimen number in each group)**	**Fold change**	***p* value**
Radvanyi et al.	Invasive lobular breast carcinoma (7) vs. normal (9)	3.135	0.005
Radvanyi et al.	Invasive mixed breast carcinoma (3) vs. normal (9)	3.158	0.012
TCGA	Invasive ductal and lobular carcinoma (3) vs. normal (61)	2.155	<0.001
TCGA	Invasive lobular breast carcnima (36) vs. normal (61)	4.414	<0.001
TCGA	Male breast carcinoma (3) vs. normal (61)	2.909	<0.001
TCGA	Invasive breast carcinoma (76) vs. normal (61)	2.984	<0.001
TCGA	Mixed lobular and ductal breast carcinoma (7) vs. normal (61)	3.476	0.005
TCGA	Invasive ductal breast carcinoma (389) vs. normal (61)	3.061	<0.001
TCGA	Mucinous breast carcinoma (4) vs. normal (61)	4.958	0.035
Karnoub et al.	Invasive ductal breast carcinoma stroma (7) vs. normal (15)	7.214	<0.001
Curtis et al.	Invasive lobular breast carcinoma (148) vs. normal (144)	2.139	<0.001

### GALNT6 was associated with poor prognosis in breast cancer patients

**Figure 2:**
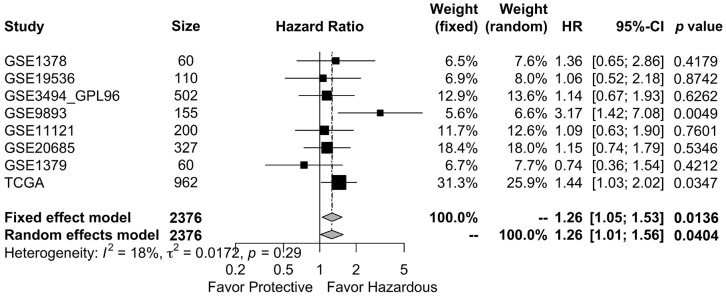
**Meta-analysis of the association between GALNT6 overexpression and OS in breast cancer studies in the SurvExpress datasets.** Eight published breast cancer studies were included for overall survival analysis. The OS pooled HR 95% CI of GALNT6 was 0.24 (0.05; 0.43) and 0.23 (0.01; 0.45) in the fixed and random effects model analyses, respectively. TE, treatment effect; seTE, standard error of TE. The dashed line on the graph represents the HR of the aggregated results. It shows the position of the meta-analysis results in each independent study.

SurvExpress, a web-based database prognostic analysis system, was used to evaluate the clinical significance of GALNT6 expression in breast cancer. First, we evaluated the prognostic role of GALNT6 in invasive breast cancer in TCGA (n = 962), which is the largest one of independent breast cancer datasets in SurvExpress. As shown in Supplementary Figure 1, patients with increased GALNT6 expression had significant shorter OS (HR = 1.44, *p* = 0.035). Furthermore, for improving the testing efficiency, we performed a meta-analysis of overall survival (OS) affected by the expression level of GALNT6 in the eight available datasets from SurvExpress. The result showed that the pooled HR (95% CI) for OS of GALNT6 was 1.26(1.05; 1.53) and 1.26 (1.01; 1.56) in the fixed- and random-effect model analyses, respectively (Figure 2 and Table 2). The p-values were 0.014 and 0.040, respectively. These data indicated that high expression of GALNT6 was significantly associated with poor OS in breast cancer. Furthermore, analysis based on TCGA database which included 1043 breast cancer samples showed that enhanced GALNT6 expression was significantly associated with advanced pN stage (*p* = 0.033) and advanced pTNM stage (with a border line significance of *p* = 0.091) (Table 3 and Supplementary Table 2). This result indicated that GALNT6 might lead to poor prognosis by promoting metastasis in breast cancer.

**Table 2:** Baseline characteristics of breast cancer studies in “SurvExpress” dataset.

**Studies**	**Author**	**Size**	**HR**	**L95CI**	**U95CI**	***p* value**
GSE1378	Ma et al.	60	1.36	0.65	2.86	0.4179
GSE19536	Enerly et al.	110	1.06	0.52	2.18	0.8742
GSE3494_GPL96	Miller et al.	502	1.14	0.67	1.93	0.6262
GSE9893	Vincent et al.	155	3.17	1.42	7.08	0.0049
GSE11121	Schmidt et al.	200	1.09	0.63	1.90	0.7601
GSE20685	Kao et al.	327	1.15	0.74	1.79	0.5346
GSE1379	Sgori et al.	60	0.74	0.36	1.54	0.4212
TCGA	TCGA-group	962	1.44	1.03	2.02	0.0347

**Table 3:** The relationship between GALNT6 expression and clinicopathological parameters.

	**GALNT6 expression of cohort from TCGA, n (%)**			**GALNT6 expression of TMA cohort, n (%)**		
	**Low (522)**	**High (521)**	***p*-value**	**Low (65)**	**High (71)**	***p*-value**
Age (years)			0.113			0.932
Median (range)	56 (26-90)	59 (26-90)		51 (31-82)	53 (29-83)	
T stage			0.266		0.802
T1	131 (25.1)	138 (26.5)		12 (18.6)	16 (22.5)	
T2	327 (62.6)	285 (54.7)		47 (71.4)	47 (66.2)	
T3	50 (9.6)	84 (16.1)		6 (10.0)	8 (11.3)	
T4	14 (2.7)	14 (2.7)				
N stage			0.033			0.004
N0	273 (52.3)	238 (45.7)		30 (41.4)	20 (28.2)	
N1-3	249 (47.7)	283 (54.3)		35 (58.6)	51 (71.8)	
TNM stage			0.091		0.029
I	87 (16.7)	92 (17.7)		4 (6.2)	6 (8.5)	
II	330 (63.2)	286 (54.9)		46 (70.8)	33 (46.5)	
III	105 (20.1)	143 (27.4)		15 (23.1)	32 (45.1)	

### Immunohistochemistry (IHC) validation of GALNT6 expression predicting poor survival in breast cancer

**Figure 3:**
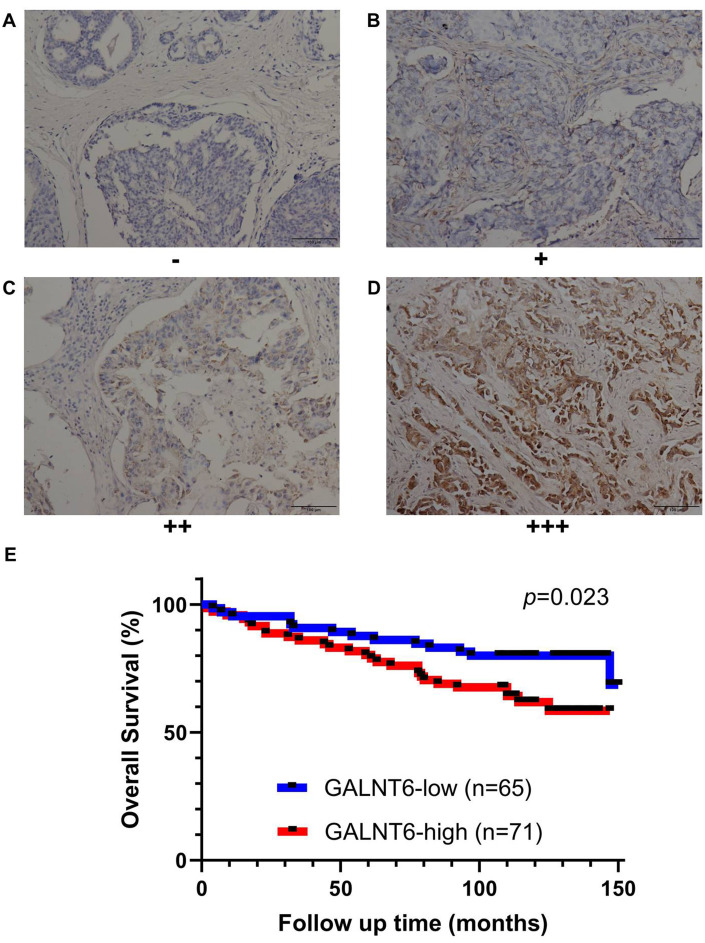
**Effect of GALNT6 expression on breast cancer survival validated by IHC based on the breast cancer tissue microarray.** (**A**–**D**) Representative images of negative, weak, moderate and strong were shown, respectively. Immunoreactivity was observed in the cytoplasm of cancer cells. (**E**) Kaplan-Meier analyses for the OS of breast patients with different levels of GALNT6.

To further validate the correlation between GALNT6 expression and prognosis in breast cancer, the expression of GALNT6 was detected in a tissue microarray of 136 breast cancer patients by IHC. GALNT6 expression was detected in the cytoplasm (Figure 3A–3D). As shown in Figure 3E, in line with relationship based on online datasets, patients with increased GALNT6 expression had significantly shorter OS (HR = 2.069, *p* = 0.023). The correlations of GALNT6 expression with clinic-pathological characteristics are further summarized in Table 3 and Supplementary Table 2. No significant difference between patients with distinct GALNT6 expression levels was observed in relation to age and pT stage. However, enhanced GALNT6 expression was significantly related to advanced pN stage (*p* = 0.004) and pTNM stage (*p* = 0.029). These data validated the role of GALNT6 in poor prognosis and metastasis of breast cancer.

### GALNT6 promoted migration and invasion in breast cancer cells

**Figure 4:**
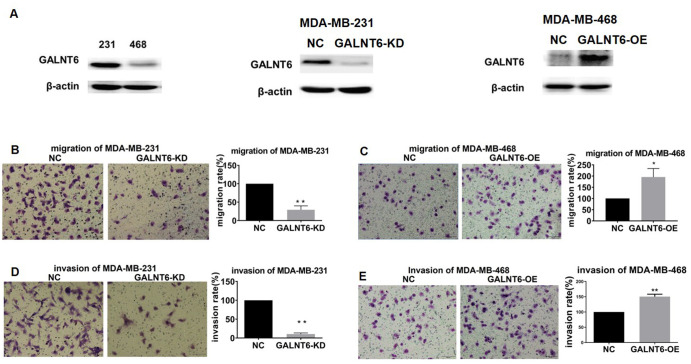
**Effect of GALNT6 on MDA-MB-231 and MDA-MB-468 cell migration and invasion.** (**A**) GALNT6 expression was detected by western blot in MDA-MB-231 and MDA-MB-468 cells, respectively (left). The efficiency of GALNT6 knock-down in MDA-MB-231 cells (middle) and overexpression in MDA-MB-468 cells (right). β-actin was used as internal control. (**B**–**E**) The effect of GALNT6-knockdown or -overexpression on migratory and invasive abilities of MDA-MB-231 and MDA-MB-468 cells.

To further investigate the role of GALNT6 in breast cancer metastasis, the effect of GALNT6 on migration and invasion was evaluated using a transwell assay in MDA-MB-231 and MDA-MB-468 cells. Since GALNT6 was highly expressed in MDA-MB-231 cells and expressed at low levels in MDA-MB-468 cells. We then knocked-down GALNT6 in MDA-MB-231 and overexpressed GALNT6 in MDA-MB-468 cells, respectively (Figure 4A). The result showed that migratory and invasive abilities decreased in GALNT6-knocked-down (KD) MDA-MB-231 cells, while they were enhanced in GALNT6-overexpressed (OE) MDA-MB-468 cells (Figure 4B–4E). These results strongly indicated that GALNT6 could promote cell migration and invasion in breast cancer cells.

### α2M was identified as a novel substrate of GALNT6 involved in metastasis promotion

**Figure 5:**
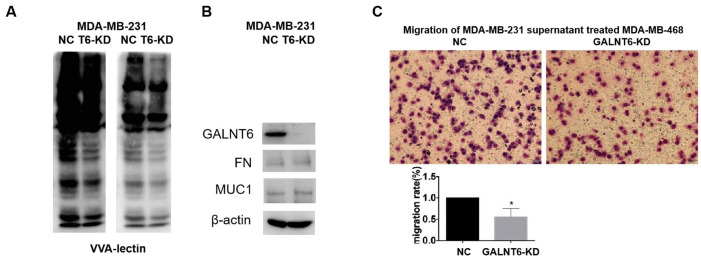
**Effect of GALNT6 mediated-mucin-type O-glycosylation of secretory proteins on breast cancer cell migration.** (**A**) VVA-lectin pull-down assay in MDA-MB-231/NC and MDA-MB-231/KD-T6 and detected by western blot at different exposure time points. (**B**) The expression of FN and MUC1 in MDA-MB-231/NC and MDA-MB-231/KD-T6 was detected by western blot. β-actin was used as internal control. (**C**) The effect of the supernatant of MDA-MB-231 on migratory ability of MDA-MB-468 cells was detected by transwell assay.

As VVA (Vicia villosa lectin) can specifically recognize a single α-N-acetylgalactosamine residue linked to serine or threonine of a polypeptide, the VVA lectin pull-down assay was used to investigate the effect of GALNT6 on mucin-type O-glycosylation in MDA-MB-231 cells. The result showed that the level of mucin-type O-glycosylation in MDA-MB-231/GALNT6-KD cells was significantly lower than that in MDA-MB-231/NC cells, indicating that GALNT6 may enhance the level of O-glycosylation on some substrates (Figure 5A). However, when detecting the known substrates of GALNT6, FN and MUC1 in MDA-MB-231 cells, only low expression levels of FN and MUC1 were observed with no band shift representing glycosylation in both MDA-MB-231/NC and MDA-MB-231/GALNT6-KD cells (Figure 5B). These results indicated that other novel substrates of GALNT6 may be involved in GALNT6-promoted metastasis in breast cancer.

Furthermore, when investigating the effect of the supernatant from MDA-MB-231 cells on the migration of MDA-MB-468 cells, it was shown that GALNT6 KD significantly reduced the migratory ability of MDA-MB-468 cells induced by the supernatant of MDA-MB-231 cells. This result suggested that new substrates secreted into the supernatant might participate in GALNT6 promoting migration and invasion (Figure 5C)*.* Moreover, to identify new substrates of GALNT6, a qualitative analysis of mucin-type glycosylomics was performed using the secreted supernatant of MDA-MB-231/GALNT6-KD and MDA-MB-231/NC cells. As shown in Table 4, AHSG, hornerin, α2M and CBFA2T2 were only identified in MDA-MB-231/NC, but not in MDA-MB-231/GALNT6-KD cells. These data suggested that these proteins may act as important substrate candidates of GALNT6 in MDA-MB-231 cells (Table 4). The levels of the four proteins in the secreted supernatant of MDA-MB-231 and MDA-MB-468 cells were verified by Western blotting. α2M and CBFA2T2 were shown to have relatively high expression levels, whilst AHSG and hornerin had low expression levels in MDA-MB-231 and MDA-MB-468 cells (Supplementary Figure 2). Although α2M was reported to be able to play an oncogenic role in cancers [23–25], it remained unclear whether α2M was involved in GALNT6 promoting metastasis, or whether GALNT6 could mediate mucin-type O-glycosylation of α2M. α2M was therefore the focus of further investigation.

**Table 4:** Substrate candidates with qualitative changes in glycosylation level before and after GALNT6 silencing in MDA-MB-231 cells.

**Protein name**	**m/z**	**Charge**	**Precurcor m/z**	**pep_ score**	**Sequence**	**Glycan**	**Position in peptide**	**Position in protein**
Alpha-2-HS-glycoprotein	719.823	4	2875.2629	23.64	LDGKFSVVYAKCDSSPDSAEDVRK	HexNAc (S)	S18	S138
Hornerin	1128.2667	4	4509.0378	11.19	MPKLLQGVITVIDVFYQYATQHGEYDTLNKAELK	Hex1HexNAc1 (T); HexNAc (T)	T10; T27	T10;T27
Alpha-2-macroglobulin	503.6593	5	2513.2603	11.08	MVSGFIPLKPTVKMLER	Hex1HexNAc1 (S); HexNAc (T)	S3; T11	S1387; T1395
CBFA2T2	853.4017	3	2557.1832	20	SSPPTMPPLPPINPGGPR	Hex1HexNAc1 (S); Hex1HexNAc1 (T)	S2;T5	S44; S47

m/z, mass-to-charge ratio.

### Mucin-type O-glycosylation of α2M promoted migration and invasion in breast cancer cells

**Figure 6:**
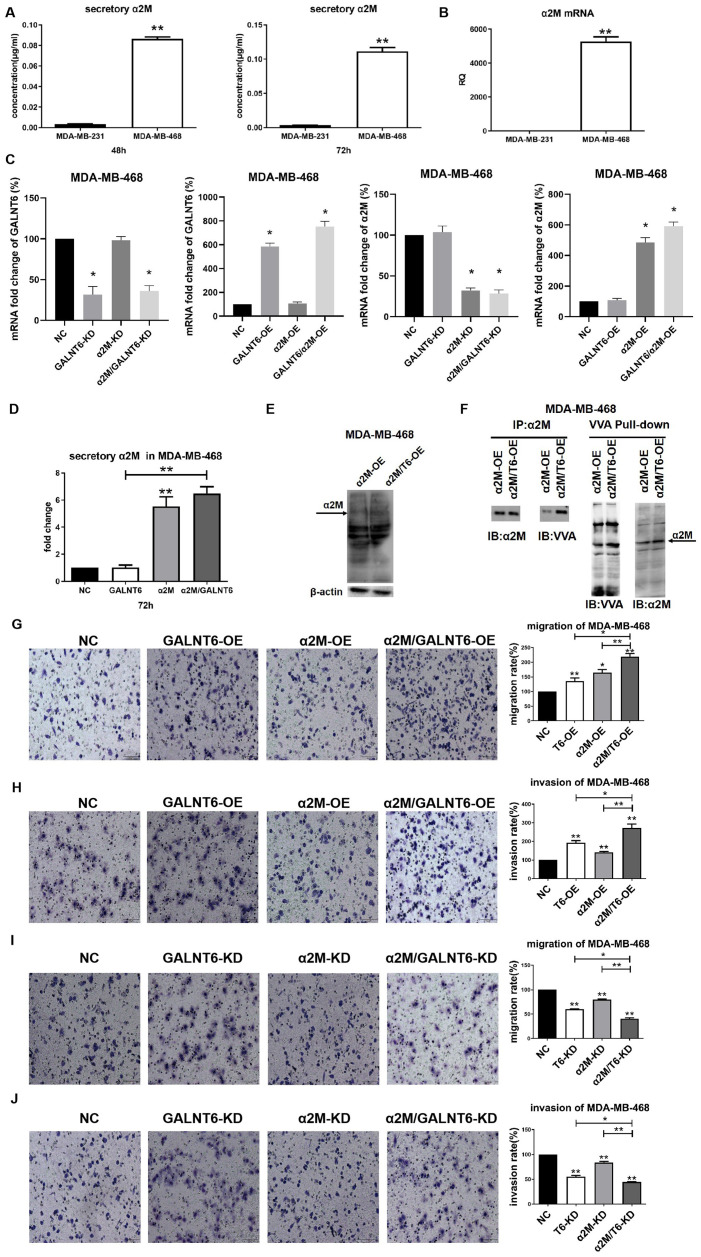
**Effect of mucin-type glycosylation of α2M on migration and invasion in breast cancer cells.** (**A**) The secretory level of α2M in MDA-MB-231 and MDA-MB-468 cells. (**B**) The mRNA level of α2M in MDA-MB-231 and MDA-MB-468 cells. (**C**) The mRNA level of α2M in MDA-MB-468/NC, MDA-MB-468/OE-GALNT6, MDA-MB-468/OE-α2M, MDA-MB-468/OE-GALNT6/α2M. (**D**) The secretory level of α2M in α2M and GALNT6 OE MDA-MB-468 cells. (**E**) The levels of O-GalNAcylation in α2M OE and α2M/GALNT6 OE MDA-MB-468 cells were verified by Western blotting using anti-O-GalNAc antibody. The arrow indicates GalNAc-conjugated α2M. β-actin was used as internal control. (**F**) Mucin-type O-glycosylation of α2M was detected by VVA lectin pull-down assay in MDA-MB-468/OE-α2M and MDA-MB-468/OE-GALNT6/α2M cells. (**G**–**J**) Migratory and invasive abilities of MDA-MB-468 cells were compared between knockdown or overexpression of GALNT6 and α2M and corresponding negative control. T6, GALNT6.

Real-time PCR and ELISA assay showed that the level of α2M was significantly higher in MDA-MB-468 than in MDA-MB-231 cells at both the transcriptional and secretory levels (Figure 6A, B). To clarify the effect of mucin-type O-glycosylation of α2M on cell migration and invasion, GALNT6 and α2M were overexpressed or knocked down in MDA-MB-468 cells (Figure 6C). α2M OE could effectively increase the secretory levels of α2M in MDA-MB-468 and MDA-MB-468/GALNT6-OE cells, whereas GALNT6 OE did not increase the secretory levels of α2M (Figure 6D). However, in simultaneous overexpression of GALNT6 and α2M MDA-MB-468, O-GalNAc levels of overall protein, including α2M (arrow) was effectively increased in comparison to MDA-MB-468/α2M-OE cells (Figure 6E). Accordingly, VVA lectin pull-down assays confirmed that the mucin-type O-glycosylation level of α2M was significantly higher in simultaneous overexpression of GALNT6 and α2M MDA-MB-468 than in MDA-MB-468/α2M-OE cells (Figure 6F). Also, α2M OE significantly enhanced the migratory and invasive abilities of MDA-MB-468 and MDA-MB-468/GALNT6-OE cells (Figure 6G, 6H). α2M KD significantly suppressed the migratory and invasive abilities of MDA-MB-468 cells and MDA-MB-468/GALNT6-OE cells (Figure 6I, 6J). Taken together, these results indicated that GALNT6 promoted migration and invasion by catalyzing mucin-type O-glycosylation of α2M.

### GALNT6 activated PI3K/Akt signaling through mucin-type O-glycosylation of α2M

**Figure 7:**
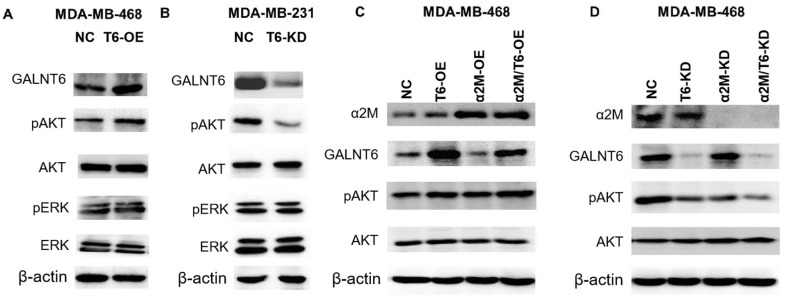
**Effect of the GALNT6-α2M axis on the activation of PI3K/Akt signaling.** (**A**) Western blot analysis of phosphorylation and baseline protein expression of Akt and ERK1/2 in MDA-MB-468/NC and MDA-MB-468/OE-GALNT6 cells. (**B**) Western blot analysis of phosphorylation and baseline protein expression of Akt and ERK1/2 in MDA-MB-231/NC and MDA-MB-231/KD-GALNT6 cells. (**C**) Western blot analysis of phosphorylation and baseline protein expression of Akt in MDA-MB-468 cells transfected with GALNT6 and/or α2M. (**D**) Western blot analysis of phosphorylation and baseline protein expression of Akt in MDA-MB-468 cells transfected with siRNA targeting GALNT6 and/or α2M. β-actin was used as internal control.

PI3K/Akt and MAPK/ERK signaling pathways were involved in GALNT6 or other GALNTs-induced cancer metastasis [26–29]. To confirm whether GALNT6-promoted metastasis is mediated by these signaling pathways, the effect of GALNT6 on the activation of Akt and ERK was detected by Western blotting. The result showed that GALNT6-OE increased Akt phosphorylation level in MDA-MB-468 cells (Figure 7A), whereas GALNT6-KD decreased Akt phosphorylation in MDA-MB-231 cells (Figure 7B). GALNT6-OE-activated PI3K/Akt signaling was further enhanced by α2M-OE (Figure 7C), whereas GALNT6-KD-inhibited PI3K/Akt signaling was further suppressed by α2M-KD (Figure 7D). However, neither KD nor OE of GALNT6 and α2M altered the phosphorylation level of ERK. These results showed that GALNT6 mediated-mucin-type O-glycosylation of α2M could activate PI3K/Akt signaling.

## DISCUSSION

In this study, we found that GALNT6 was associated with poor prognosis and promoted metastasis in breast cancer. GALNT6 was shown to promote metastasis of breast cancer cells by enhancing mucin-type O-glycosylation, and α2M was identified as a novel substrate of GALNT6 in the secretory supernatant of breast cancer cells. Also, GALNT6 was confirmed to promote metastasis by catalyzing O-glycosylation of α2M and activating the downstream AKT signaling pathway.

According to a previous study, the rate of bone marrow dissemination was 54.5% in GALNT6-positive patients, but only 4.3% in GALNT6-negative patients, suggesting that GALNT6 could be used as a biomarker of breast cancer metastasis [19]. Our results based on the Oncomine database showed that GALNT6 was up-regulated in 11 out of 45 breast cancer analyses comparing with normal tissues, indicating that GALNT6 might play an important role in breast cancer progression. The increase in GALNT6 expression and its consistency in different datasets are almost the most significant in all malignancies. Although online data has limitations due to the heterogeneity of malignant tumors, the limitations of high-throughput technology, and relatively small sample size of a single study, the integrated multiple central data greatly increases the convincingness of the results. TCGA-based survival analysis and further meta-analysis of multiple studies from the SurvExpress online dataset showed GALNT6 expression was significantly positively correlated with poor OS in breast cancer. Compared with using a single dataset, our meta-analysis effectively improves test efficiency and expands the applicability of the results to the general population. We further validated the prognostic role of GALNT6 by IHC in breast cancer in tissue specimens. These results confirmed the importance of high expression of GALNT6 in promoting poor prognosis of breast cancer. Enhanced GALNT6 expression was significantly correlated with advanced pN stage, indicating that GALNT6 might lead to poor prognosis by promoting metastasis. Subsequently, our cytology experiment confirmed that GALNT6 could promote migration and invasion of breast cancer cells. Therefore, all our results clearly indicated that GALNT6 is a tumor promoter gene in breast cancer.

GALNT6 is known to function as an enzyme in the catalysis of mucin-type O-glycosylation of substrates. Our VVA pull-down assay results showed that GALNT6 increased the level of mucin-type O-glycosylation. Previous studies have reported that MUC1 and FN are classic mucin-type O-glycosylation substrates of GALNT6 which can promote the malignant phenotype of breast cancer cells [20, 21]. However, in this study, only low expressions of MUC1 and FN were observed, and no mucin-type O-glycosylation changes were detected in MDA-MB-231 cells. Therefore, we speculated that other substrates may be involved in the GALNT6-meditated promotion of breast cancer metastasis.

Mucin-type O-glycosylation mainly occurs in membrane proteins on the cell surface and secreted proteins in the extracellular matrix [30]. Although several studies have revealed that GALNTs promote malignant behavior of cancer by catalyzing different substrates [31], few reports have investigated GALNTs substrates in secreted proteins. Through the qualitative mucin-type glycosylomics analysis, we identified AHSG, hornerin, α2M and CBFA2T2 as novel secretory substrates of GALNT6. Alpha-2-heremans schmid glycoprotein (AHSG), also known as fetuin A, is a glycoprotein mainly secreted by the liver [32] that is also produced by cancer cells [33]. Hornerin is an S100 protein family member and a type of calcium binding protein [34]. α2M is mainly synthesized in the liver and is a typical pan-proteinase inhibitor in humans [35]. Myeloid translocation gene-related 1 (MTGR1, also known as CBFA2T2), is a transcriptional corepressor in the myeloid translocation gene family [36]. Aberrant expression levels and dysfunctions of the four substrates are closely correlated with proliferation, apoptosis, metastasis and angiogenesis in different types of cancers [35, 37–39]. As α2M and CBFA2T2 showed relatively high expression, whilst AHSG and hornerin showed extremely low expression in MDA-MB-231 and MDA-MB-468 cells, we speculated that α2M and CBFA2T2 may play key roles in GALNT6-mediated metastasis of breast cancer. Considering the oncogenic role of GALNT6 in promoting metastasis, α2M was selected for further investigation as an important substrate in breast cancer metastasis in current study. Moreover, the identification of GALNT6-mediated mucin-type O-glycosylation of CBFA2T2 and the role of CBFA2T2 in GALNT6-mediated progression should be subject to further investigation in the future. Our data have opened up a new idea for revealing the role of mucin-type O-glycosylation in malignant tumors.

Overexpression of α2M has been detected in different types of cancers and functions as a regulator of many signaling pathways [40–44]. In our study, α2M was identified as one of the most important mucin-type O-glycosylated proteins regulated by GALNT6 from the qualitative glycosylation analysis. We found that GALNT6 overexpression did not affect the secretory levels of α2M, but instead significantly increased the levels of mucin-type O-glycosylation of α2M. Furthermore, the transcriptional expression and secretory levels of α2M were relatively lower in MDA-MB-231 cells with higher GALNT6 expression, compared with the MDA-MB-468 cells with lower GALNT6 expression. These results may be because the efficacy of α2M is not only related to the secretory level, but also closely related to the level of mucin-type O-glycosylation.

Activation of intracellular PI3K/Akt is a key signaling pathway of various secretory proteins in the maintenance of the cancer malignant biological behavior [45]. Previous studies reported that silencing of GALNT2 could inhibit insulin-induced insulin receptor activation and Akt phosphorylation in hepatoma cells [46], whereas overexpression of GALNT2 in oral cancer cells could promote EGF-induced phosphorylation of EGFR and Akt [47]. In addition, α2M was reported to bind to the GRP78 receptor on the surface of the cell membrane, activating the IGFR and mTOR signaling pathways in prostate cancer, suggesting the involvement of the AKT/mTOR pathway in α2M-mediated cancer development [42, 43]. Consistent with the studies above, we found that GALNT6 could activate PI3K/Akt signaling, and knockdown of α2M attenuated the activation of PI3K/Akt signaling induced by GALNT6, suggesting that GALNT6 activated PI3K/Akt signaling through mucin-type O-glycosylation of α2M. However, it is unclear whether α2M activated PI3K/Akt pathway is directly or through binding to specific cell membrane protein receptors. Further investigation is required to decipher the precise mechanism of action in this pathway.

**Figure 8:**
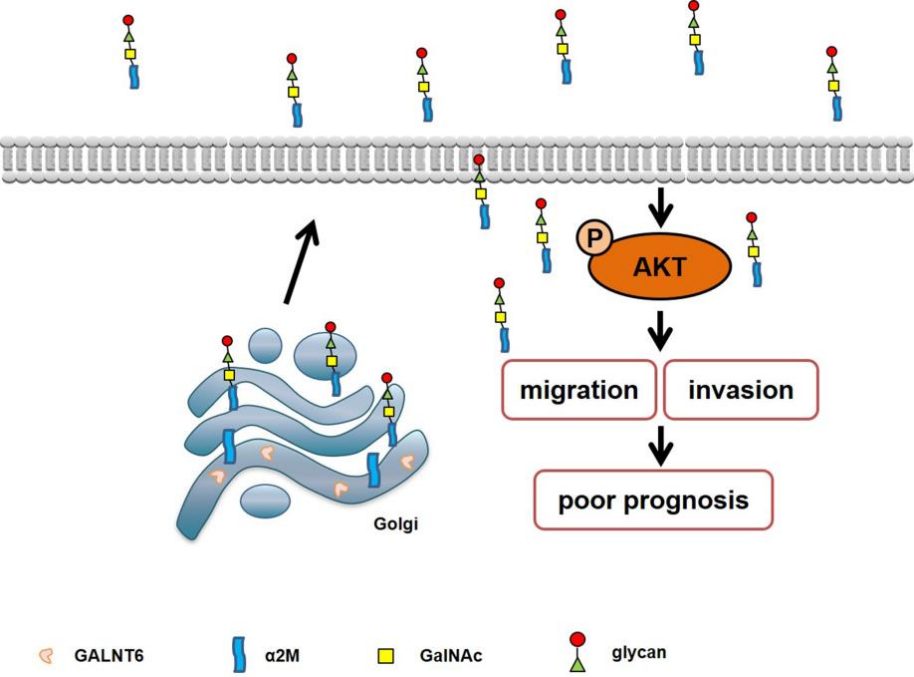
**Schematic diagram of the mechanism of GALNT6-promoted metastasis through increasing mucin-type O-glycosylation of α2M in breast cancer.**

In summary, our study demonstrates that GALNT6 can promote metastasis by increasing mucin-type O-glycosylation of α2M, and activating the downstream PI3K/Akt signaling pathway in breast cancer cells (Figure 8). These findings provide a new idea for studying the role of GALNT6 in promoting a malignant phenotype and poor prognosis of breast cancer.

## MATERIALS AND METHODS

### Oncomine database analysis

ONCOMINE datasets (http://www.oncomine.org) is an online cancer microarray database [48]. The mRNA level of GALNT6 was determined using ONCOMINE. The RNA levels of clinical specimens from cancer and normal control datasets were compared. The fold change was defined as 2, and Student’s *t*-test was used to generate *p*-value. A *p*-value of < 0.05 was identified as statistically significant.

### Survival express

SurvExpress is an online biomarker validation tool (http://bioinformatica.mty.itesm.mx:8080/Biomatec/SurvivaX.jsp) that was used to investigate the prognostic value of GALNT6 [49]. Eight published breast cancer studies with a total of 2,376 samples were selected to investigate OS in the meta-analysis. Table 2 shows the cohorts list for meta-analysis with the default settings showing the study name, sample size, HR and 95% CI.

### TCGA data acquisition and screening

The TCGA datasets provided RNASeqV2 data on 1,095 breast cancer cases, which were obtained by searching for BRCA cases from the TCGA website (http://cancergenome.nih.gov/). A total of 52 cases were excluded because of no stage information (11 cases), stage X (13 cases), stage IV (20 cases) or Nx (8 cases). The expression data of the GALNT6 gene were collected for each eligible case and divided into high- and low-expression groups. The cut-off was set to 50%.

### Patients and tissue specimens in the tissue microarray

A tissue microarray containing 136 primary breast cancer tissues, was obtained from the Outdo Biotech Co., Ltd (Shanghai, China) and was approved for use in this study (YB M-05-01) by the Ethical Committee of Outdo Biotech Co., Ltd. All patients gave informed consent for voluntary participation and agreed to report individual patient data. Clinical medical records and follow-up data of 136 primary breast cancer patients subjected to resection between January 1, 2001 and August 31, 2004 were reviewed. This report included follow-up data up to July 31, 2013, with a median follow-up period of 114.5 months (ranging from 2 to 150 months). OS was defined on the period from the data of surgery to death or the last follow-up date.

### Immunohistochemistry (IHC)

IHC was performed as previously described [50]. Anti-human GALNT6 antibody was obtained from Sigma-Aldrich (St. Louis, MO, USA) and used at a 1:200 dilution. All specimens were independently scored by two observers in a blinded manner. Quantitative analysis was performed based on the intensity of staining and percentage of the positive cells. The staining intensity of GALNT6 was scored as 0 for negative, 1 for weak, 2 for moderate and 3 for strong. The extent of staining was scored as 0 to 4 for 0%, 1-25%, 26-50%, 51-75%, and 76-100%, respectively. The staining score was defined as the sum of the scores of intensity and extent. Staining scores from 0-4 were classified as low expression, and 5-7 as high expression for dichotomous modeling.

### Cell cultures

Human breast cancer cell lines, MDA-MB-468 and MDA-MB-231, were obtained from the Type Culture Collection of the Chinese Academy of Sciences (Shanghai, China). Cells were maintained in L15 medium (Gibco, Gaithersburg, MD, USA) supplemented with 10% fetal bovine serum. All cells were cultured at 37°C in a humidified atmosphere containing 5% CO_2_.

### Lentiviral transduction and cell transfection in breast cancer cell lines

Lentiviral vector of LV-GALNT6-RNAi for GALNT6 knockdown (KD) and negative control vector hU6-MCS-CMV-EGFP (KD-NC), and LV-GALNT6 for GALNT6 overexpression (OE) and mock vector Ubi-MCS-3FLAG-CMV-EGFP (OE-NC) were obtained from GeneChem Co., Ltd. (Shanghai, China). Prior to transduction, MDA-MB-231 and MDA-MB-468 cells were seeded at 1×10^5^ and 2×10^5^ cells per well in a 6-well plate overnight, respectively. Both of the two cell lines were transducted at a multiplicity of infection (MOI) of 10. Polybrene and Enhanced Infection Solution (ENi.S.) were used as the transduction enhancer. The lentivirus transduction procedures were performed according to the manufacturer’s instruction. Pooled clones with infection efficiency above 85% were harvested for the subsequent cellular and molecular biology experiments. The efficiency of knockdown and overexpression was determined by Western blotting.

Specific siRNAs, the pcDNA encodes GALNT6 and their corresponding control was transfected employing the Lipofectamine 2000 reagent (Thermo Fisher Scientific) following the manufacturer’s instructions. After 48h transfection, the expression of target protein and gene were evaluated by real-time PCR and Western blotting.

The sequences of siRNAs are shown below: GALNT6: 5’-GCAGACUCUGUUCUCCAUAtt-3’ (sence), 5’-UAUGGAGAACAGAGUCUGCtt-3’ (antisence); α2M: 5’-GCCGAUCCUUCUCCGUGCCtt-3’ (sence), 5’-CUCGCAGUACAUUGACAGCtt-3’ (antisence); negative control: 5’-UUCUCCGAACGUGUCACGUtt-3’ (sence), 5’-ACGUGACACGUUCGGAGAAtt-3’ (antisence).

### RNA extraction and quantitative real-time polymerase chain reaction (qRT-PCR)

Total RNA was extracted from cell lines as described previously [51]. First strand cDNA was synthesized with PrimeScript^®^ RT Master Mix (TaKaRa, Japan), according to the manufacturer’s protocols. After reverse transcription of total RNA, qRT-PCR was carried out to determine the expression levels of α2M using SYBR^®^ Advantage^®^ qPCR Premix (TaKaRa, Japan) on the ABI 7500 Sequence Detection System (Applied Biosystems, Foster, CA). The sequences for α2M were 5’-AAGGTCCAGGCCCACTGAAG-3’ (forward) and 5’-CAGTTCAGGTGACAGAGGCTCAA-3’ (reverse), and the sequences for internal reference gene 18s were 5’-CCCGGGGAGGTAGTGACGAAAAAT-3’ (forward) and 5’-CGCCCGCCCGCTCCCAAGAT-3’ (reverse). The PCR conditions were 30 s at 95°C, followed by 45 cycles at 95°C for 5 s, and 58°C for 34 s. Data were analyzed using the Applied Biosystems 7500 software program (version 2.3) with automatic Ct setting for adapting baseline and threshold for Ct determination. The threshold cycle and 2^-ΔΔCt^ method were used for calculating the relative amounts of the target mRNA.

### Enzyme linked immunosorbent assay (ELISA)

MDA-MB-231 and MDA-MB-468 cell lines, or GALNT6 and/or α2M gene overexpression and corresponding control MDA-MB-468 cell lines were seeded in 6-well plates at a density of 2×10^6^/well. After being cultured in serum-free L15 medium for 48 or 72 hours, cells and corresponding supernatants were collected, respectively. Concentrations of α2M in supernatants were measured using an ELISA kit (Abcam, MA, USA) according to the manufacturer’s protocols, and were adjusted according to the total protein amount in cell lysates.

### Cell migration and invasion assays

For migration assays, MDA-MB-231/NC or MDA-MB-231/GALNT6-KD cells and MDA-MB-468/NC or MDA-MB-468/GALNT6-OE cells (5×10^4^) in serum free L15 medium were seeded into the upper chamber of the transwell (8 μm pores; Costar, Cambridge, MA, USA), and 2% FBS in L15 was added to the lower chamber. After incubation at 37°C in 5% CO_2_ for 24 h, cells were fixed with cold ethanol and stained with Wright-Giemsa. For invasion assays, chambers were pre-coated with 40 μl of Matrigel^TM^ at a concentration of 0.5 mg/ml (BD Biosciences, Franklin Lakes, NJ, USA) before cell seeding. All other processes were identical to those used in the migration assays.

To investigate the effect of GALNT6-mediated mucin-type O-glycosylation of secretory proteins on the migration of MDA-MB-468 cells, the media of MDA-MB-231/GALNT6-KD and MDA-MB-231/NC cells cultured for 48 h were collected and added to the lower chamber. MDA-MB-468 cells were seeded into the upper chamber of the transwell plate. The migratory or invasive cells on the underside of the filter were photographed. Cells from five random fields of each well were counted to determine the average number of migratory or invasive cells.

### Western blotting

Western blotting was performed as described previously [52]. The primary antibodies were obtained as follows: anti-GALNT6 was purchased from Sigma-Aldrich (St. Louis, MO, USA); anti-α2M was from Abcam (MA, USA); anti-GalNAc was from Novus (Medtronic, MN, USA); anti-β-actin was from Santa Cruz Biotechnology (CA, USA); anti-Akt, anti-p-Akt, anti-ERK, and anti-p-ERK were obtained from Cell Signaling Technology (Beverly, MA, USA). Enhanced chemi-luminescence reagent (SuperSignaling Western Pico Chemiluminescent Substrate; Pierce, Rockford, IL, USA) and the electrophoresis gel imaging analysis system (DNR Bio-Imaging Systems, Jerusalem, Israel) were used to visualize the signals and analyze the proteins. Final results were analyzed using NIH Image J software.

### VVA lectin pull-down assay for Mucin-type O-glycosylated proteins

Cell lysates containing 600 μg of protein were incubated with 4 μg of biotinylated lectin VVA (Vector Laboratories, CA, USA) for 3 h at 4 °C. After adding 20 μl of streptavidin-agarose (ThermoFisher, MA, USA), the mixture of samples was further incubated for an additional 2 h at 4 °C with rotation. Glycoprotein/lectin complexes were collected by brief centrifugation (1400 rpm, 5 min), and washed 3 times with lysis buffer, followed by one wash with PBS. Glycoproteins were released from the complexes by boiling in 30-50 μl of SDS-PAGE sample buffers. Western blotting was conducted to detect GalNAc-conjugated proteins.

### Glycosylomics

Secretory proteins enriched from the culture medium of MDA-MB-231/GALNT6-KD and MDA-MB-231/NC cells were digested with trypsin. After removing N-glycosylation by treatment with glycosidase, O-GalNAc affinity enrichment was conducted by hydrophilic chromatography. High sensitivity and resolution LC-MS analysis was performed on the affinity-enriched peptides.

### Statistical analysis

For continuous variables (such as age), a Welch’s two-sample *t*-test was performed to generate *p* values. Categorical variables (such as T stage, N stage, TNM stage, *etc*.) were compared using the *χ^2^* test or Fisher’s exact test. *P* value < 0.05 was considered to be statistically significant.

## Supplementary Material

Supplementary Figures

Supplementary Tables
