# Supplementary material for: GALNT6 promotes breast cancer metastasis by increasing mucin-type O-glycosylation of α2M
Source: Aging (Albany NY). 2020 Jun 18;12(12):11794–811. doi: 10.18632/aging.103349 (PMC7343513; doi:10.18632/aging.103349)
Supplement: Supplementary Figures [file aging-12-103349-s001..pdf]

## SUPPLEMENTARY FIGURES

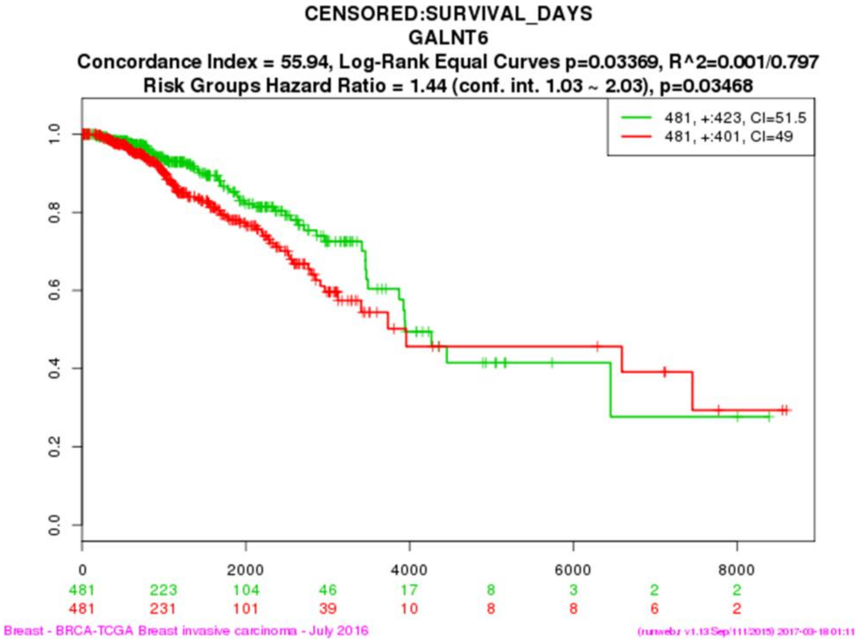


**Supplementary Figure 1. Kaplan-Meier curve for the OS of invasive breast cancer patients with different levels of GALNT6 based on TCGA in SurvExpress.**

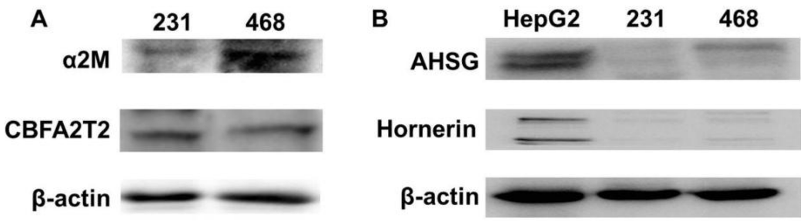


**Supplementary Figure 2. Protein levels of AHSG, hornerin, α2M and CBFA2T2 in the secreted supernatants of MDA-MB-231 and MDA-MB-468 cells detected by Western blotting.** (**A**) α2M and CBFA2T2; (**B**) AHSG and hornerin. HepG2 was used as positive control for AHSG and hornerin. β-actin was used as internal control.
