# Supplementary material for: GALNT6 promotes breast cancer metastasis by increasing mucin-type O-glycosylation of α2M
Source: Aging (Albany NY). 2020 Jun 18;12(12):11794–811. doi: 10.18632/aging.103349 (PMC7343513; doi:10.18632/aging.103349)
Supplement: Supplementary Tables [file aging-12-103349-s002..pdf]

SUPPLEMENTARY TABLES

Supplementary Table 1. Summary of numbers of up-regulated, down-regulated and nonsignificant analyses and samples for different types of cancers in Oncomine.

	Up-regulated		Down-regulated		Nonsignificant		Total	
	Analyses	samples	Analyses	samples	Analyses	samples	Analyses	samples
Bladder Cancer	0	0	0	0	-	-	-	-
Brain and CNS Cancer	1	101	5	213	18	2413	24	2727
Breast Cancer	11	2814	0	0	34	2026	45	4840
Cervical Cancer	2	150	0	0	8	386	10	536
Colorectal Cancer	22	886	0	0	10	1069	32	1955
Esophageal Cancer	6	224	0	0	3	238	9	462
Gastric Cancer	0	0	3	69	17	1205	20	1274
Head and Neck Cancer	5	255	0	0	22	1970	27	2225
Kidney Cancer	0	0	1	67	19	1306	20	1373
Leukemia	4	2398	0	0	17	905	21	3303
Liver Cancer	0	0	0	0	-	-	-	-
Lung Cancer	3	428	0	0	17	2101	20	2529
Lymphoma	0	0	2	200	19	237	23	437
Melanoma	0	0	0	0	-	-	-	-
Myeloma	0	0	0	0	-	-	-	-
Other Cancer	11	239	0	0	15	1056	26	1295
Ovarian Cancer	2	248	0	0	10	1915	12	2163
Pancreatic Cancer	2	88	0	0	8	307	10	395
Prostate Cancer	0	0	1	19	9	924	10	943
Sarcoma	0	0	1	90	17	354	18	444

Supplementary Table 2. Characteristics of the TCGA breast cancer study cohort and the tissue microarray (TMA) cohort.

Characteristics	TCGA cohort (N=1043)		Cohort from the TMA (N=136)	
	Number	%	Number	%
Age,median (range)	58	26-90	52	29-83
**Gender**				
Female	1043	100.0	136	100.0
**T stage**				
T1	93	34.1	28	20.6
T2	156	57.1	94	69.1
T3	24	8.8	14	10.3
**N stage**				
N0	186	68.1	50	36.8
N1-3	87	31.9	86	63.2
**TNM stage**				
I	166	60.8	10	7.4
II	69	25.3	79	58.1
III	38	13.9	47	34.6
